# Transcriptome sequencing and *de novo* annotation of the critically endangered Adriatic sturgeon

**DOI:** 10.1186/1471-2164-14-407

**Published:** 2013-06-18

**Authors:** Michele Vidotto, Alessandro Grapputo, Elisa Boscari, Federica Barbisan, Alessandro Coppe, Gilberto Grandi, Abhishek Kumar, Leonardo Congiu

**Affiliations:** 1 Department of Biology, University of Padova, Padova, Via G. Colombo 3, I-35131, Italy; 2 Department of Biology, University of Ferrara, Ferrara, Via L.Borsari 46, I-44100, Italy; 3 Abteilung für Botanische Genetik und Molekularbiologie Botanisches, Institut und Botanischer Garten, Christian-Albrechts-Universität, Kiel, Germany

## Abstract

**Background:**

Sturgeons are a group of Condrostean fish with very high evolutionary, economical and conservation interest. The eggs of these living fossils represent one of the most high prized foods of animal origin. The intense fishing pressure on wild stocks to harvest caviar has caused in the last decades a dramatic decline of their distribution and abundance leading the International Union for Conservation of Nature to list them as the more endangered group of species. As a direct consequence, world-wide efforts have been made to develop sturgeon aquaculture programmes for caviar production. In this context, the characterization of the genes involved in sex determination could provide relevant information for the selective farming of the more profitable females.

**Results:**

The 454 sequencing of two cDNA libraries from the gonads and brain of one male and one female full-sib *A. naccarii,* yielded 182,066 and 167,776 reads respectively, which, after strict quality control, were iterative assembled into more than 55,000 high quality ESTs. The average per-base coverage reached by assembling the two libraries was 4X. The multi-step annotation process resulted in 16% successfully annotated sequences with GO terms. We screened the transcriptome for 32 sex-related genes and highlighted 7 genes that are potentially specifically expressed, 5 in male and 2 in females, at the first life stage at which sex is histologically identifiable. In addition we identified 21,791 putative EST-linked SNPs and 5,295 SSRs.

**Conclusions:**

This study represents the first large massive release of sturgeon transcriptome information that we organized into the public database *AnaccariiBase*, which is freely available at http://compgen.bio.unipd.it/anaccariibase/. This transcriptomic data represents an important source of information for further studies on sturgeon species. The hundreds of putative EST-linked molecular makers discovered in this study will be invaluable for sturgeon reintroduction and breeding programs.

## Background

Sturgeons (order: Acipenseriformes, infraclass: Chondrostei) are a very ancient fish group distributed in the Palearctic hemisphere with about 25 species, most of which are considered to be on the brink of extinction [1]. Their conservation importance has led to the inclusion of all the species in the red list of the International Union for the Conservation of Nature (IUCN) and to commercial restrictions under the Convention for International Trading of Endangered Species (CITES). These fish are also interesting from a biological standpoint, presenting peculiarities that make the characterization of their transcriptome worthwhile. Often referred to as living fossils, sturgeons very ancient separation from teleosts occurred over 250 Mya [2], placing them in a key phylogenetic position for evolutionary studies on vertebrates. A second aspect of interest is related to the ploidy of sturgeons. Different species are characterized by different degrees of ploidy which are the result of multiple and independent duplication events [3-5].

Sturgeon species can be divided into two main groups based on their number of chromosomes: the first having approximately 120 and the second approximately 240 chromosomes. The level of ploidy to be ascribed to each chromosome number is still being debated. Some authors consider species of the two groups to be diploid and tetraploid respectively [6] while others attribute the tetra/octoploid condition to the same groups [7]. In this paper we characterised the transcriptome of the Adriatic sturgeon (*Acipenser naccarii*) which belong to the 240 chromosomes group [8]. A detailed transcriptome investigation of a polyploid sturgeon species will become crucial to assess the functional reduction of ploidy once the genome will be sequenced or once either the trancriptome of a 120 chromosomes species will be available. Following a polyploidization event, in fact, the redundant genetic material experiences a functional reduction which can be monitored by analysing the transcribed part of the genome [9].

The high economic value of these animals adds another aspect to the characterisation of the transcriptome of a sturgeon species. Sturgeon eggs, sold as caviar, are one of the most valuable products in international food trade [10] and their very high monetary value is the main cause of the extremely-endangered status of most sturgeon species. In response to the rapid decline of natural populations, aquaculture production of caviar is rapidly increasing. One of the main problems for aquaculture caviar producers is that 50% of the animals are profitless males which need to be discarded from production as quickly as possible to minimise expenditure and maximise space. However, sex discrimination in sturgeon farming for caviar production can only be performed by ultrasound analysis after 4 or 5 years. The rearing of males can, thus, represent up to 30% of total farming costs [11]. A genetic identification of the sexes at an early life stage based on PCR techniques could, therefore, contribute to lowering the costs of caviar production in aquaculture and have knock-on effects in both farming and conservation. Aquaculture activity would significantly benefit from this possibility and poaching on natural populations would consequently be reduced.

There are good indications that sex is genetically determined in sturgeon [11,12] however, genomic screening performed with the aim of identifying a sex marker has not, as yet, yielded satisfactory results [11,13]. Knowledge of which genes are involved in sex differentiation in sturgeons is limited and analyses at the transcriptome level of the expressed genes at the first stage at which sex can be histologically determined could contribute to expanding the knowledge base.

In a March 2010 press release, IUCN identified sturgeons as the world's most endangered group of animals with 85% of the species being at risk of extinction. For the very low numbers of wild breeders, future restoration efforts must rely on *ex situ* conservation strategies through the setup of long-term breeding programs. The availability of a high number of genetic markers to guarantee adequate genetic support to releasing activities, through parental allocation and traceability of the hatchery of origin, become important in this context. Moreover, the availability of EST-linked markers yielded by transcriptome characterisation may provide a suitable tool for the identification of footprints of selective pressures in the released stocks, due to natural or anthropogenic stress.

The present paper reports the first characterisation of the Adriatic sturgeon transcriptome obtained by 454 titanium sequencing. We provide the results of the comparison of one male and one female library. Finally we characterized microsatellite and SNPs loci to be employed for conservation purposes.

The results of this characterization have been organized in a public database which represents to our knowledge the first large amount of information of a sturgeon transcriptome.

## Results and discussion

### Cleaning and assembly

Two one-quarter picotiter plates of a 454 FLX sequencing run generated 154,882 and 176,703 reads from the *A. naccarii* male (cDNA3) and female (cDNA4) respectively. FastQC [14] overview of raw sequences showed that mean per-base quality remains above 24 for the first 350 bp and, thereafter, drops rapidly towards the end of the reads (data not shown). The cleaning process was passed by 99% of the reads from each library, yielding a total of 110.25 Mbp of cleaned sequences with an average length of 336 bp and mean Phred quality of 28. The main features of the sequences that passed the preprocessing step are summarized in Table 1 while their length distribution is plotted in Additional file 1. The mean GC content calculated for the whole dataset was 37.92%. GC content across sequence length follows a normal distribution thus discarding the hypothesis that systematic bias was present (data not shown). As expected, more than 50% of the total sequences (121,467 sequences) were 400 bp or longer.

**Table 1:** **Statistics of reads preprocessing for *A. naccarii *libraries**

**Category**	**Male (cDNA3)**	**Female (cDNA4)**	**Total**
Total number of raw reads	154,882	176,703	331,585
Total number of cleaned reads	153,215	175,198	328,413
Percentage of cleaned reads	99.00	99.00	99.00
Median length (bp)	376	354	365
Average GC content in percentage	37.77	38.06	37.92
Total length of cleaned reads (Mb)	52.91	57.34	110.25
Average phred quality of cleaned reads	28	28	28

The first round of MIRA assembled 256,738 reads (77.43% of the total cleaned reads) into 44,232 contigs and 16,593 singletons. The first assembly resulted in 27.62 Mbp of total consensus, composed of 60,825 sequences with an average length of 454.14 bp, average Phred quality of 39, a mean GC content of 38.47% and an average coverage of 4.22 reads. More details about the generated contigs and singletons are reported in Table 2. In the second round MIRA reassembled 6,242 contigs (14%) and 3,504 singletons (21%) from the previous assembly into 4,203 metacontigs, with an average coverage of 2.32 sequence/metacontig (Table 3).

**Table 2:** **Contigs and singletons summary statistics for first round and final assembly by MIRA of *A. naccarii *transcriptome**

	**Round 1**	**Final assembly**
Reads assembled (#)	256,738	256,738
Reads assembled (%)	77.43	77.43
***Total contigs (#)***	***44,232***	***42,193***
Contigs (%)	72.72	76.32
Total contigs length (Mb)	22.64	21.87
Average contigs length (bp)	511.89	518.29
Average contigs GC content (%)	38.49	38.83
Average contigs quality (phred)	43	43
Average contigs coverage (bp/position)	3.2	4.09
***Total singletons (#)***	***16,593***	***13,089***
Singletons (%)	27.28	23.68
Total singleton length (Mb)	4.98	3.91
Average singleton length (bp)	300.19	298.55
Average singleton GC content (%)	38.39	38.46
Average singleton quality (phred)	28	28

**Table 3:** Metacontigs summary statistics after the second round assembly by MIRA

	**Round 2**
***Total metacontigs (#)***	4,203
Reassembled contigs (#)	6,242
Percentage of reassembled contigs	14.11
Reassembled singletons (#)	3,504
Percentage of reassembled singletons	21.12
Total consensus metacontig (Mb)	2.95
Metacontig average length (bp)	700.94
Percentage of metacontig average GC content	38.83
Metacontig average consensus quality (phred)	45
Metacontig average coverage (bp/position)	1.66

**Figure 1:**
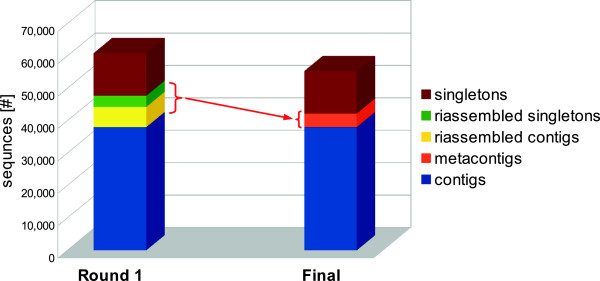
**Redundancy reduction after two assembly rounds with MIRA for *A. naccarii *data.** Graphical representation of the contigs and singletons built in the first assembly round, which were re-assembled as metacontigs in the second round, and then joined to get the final assembly.

Finally the two assembly runs were merged giving a total of 55,282 sequences, 42,193 contigs plus metacontigs (21.87 Mbp) and 13,089 singletons (3.91 Mbp). This resulted in a 9.11% sequence reduction compared to the first assembly as clearly illustrated by Figure 1. Overall, the sequences of this final dataset were characterized by a mean length of 466 bp, an average Phred quality of 40 and a mean coverage of 4.64 reads. GC content remained the same as in the first assembly (details relating to contigs and singletons are shown in Table 2). Changes in length and quality distribution of contigs from the first to the second round assembly are shown in Additional file 2 and Additional file 3 respectively.

We performed the iterative assembly process being aware that some degree of assembly accuracy is lost. In fact, by forcing MIRA to resolve ambiguous positions by choosing a consensus, the probability of losing rare transcriptional variants is increased. However, two assembly cycles were performed for two reasons: 1) we were interested in having a general overview of genes expressed in *A. naccarii* by minimizing redundancy, and 2) information on rare variants can be traced back, realigning all the original reads on the corresponding contigs. After assembly, all reads of origin were aligned against belonging contigs and metacontigs, obtaining a multiple alignment for each of them. The distribution of the average coverage observed in the contigs and metacontigs from the first and final assemblies are reported in Additional file 4. Pair-wise relationships between sequence length, number of reads per contig and average sequence quality after the two assemblies are shown in Additional file 5. All contigs and cleaned reads are provided within the AnaccariiBase database, available at the web page: http://compgen.bio.unipd.it/anaccariibase/. From here on, we will no longer make any distinction between contigs and metacontigs and both will be indicated simply as contigs.

### Functional annotations

*De novo* annotation of *A. naccarii* transcriptome was performed with multi-step procedure starting from similarity search against gender specific nucleotide sequences, main protein and nucleotide databases, full transcribed and protein sequences from other fishes in Ensembl database.

### BLAST against sequences available from the genus *Acipenser*

The comparison of *A. naccarii* sequences with 6,088 ESTs for the genus *Acipenser* already available revealed 8,804 *A. naccarii* contigs (15.93%) matching 2,047 different subjects (33.62%). The limited percentage of matching sequences can probably be ascribed to the different tissues of origin: gonad and brain in the Adriatic sturgeon, and mainly pituitary gland, skin and spleen in the reference database.

### BLASTX against the main protein sequence databases

**Figure 2:**
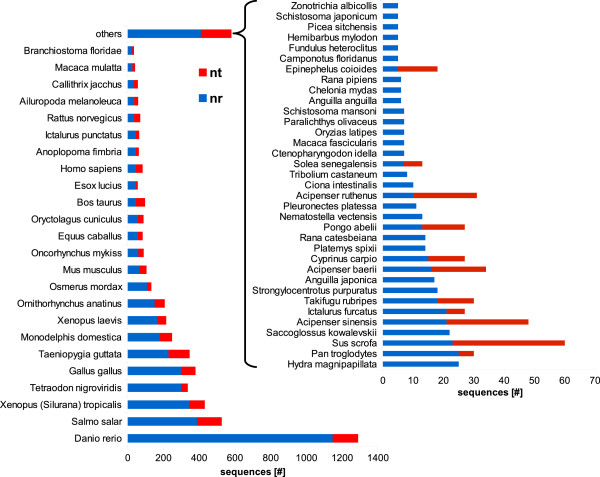
**Taxonomic classification of *A. naccarii *contig annotations.** Assignment of annotations obtained from BLASTX and BLASTN comparisons (e-value 1e-03) of contigs against NCBI nr and nt databases to different species was performed with MEGAN 4, based on the absolute best BLAST hits. The bar chart shows contigs annotated with the 24 more-represented species in annotations from nr. The contribution of annotations from nt, for the same species, is marked in red. “Others” includes the 34 species less represented in nr annotations.

The comparison of contigs and singletons to the NCBI non-redundant protein database (nr) using BLASTX, came out with 9,850 contigs and 2,339 singletons (22.05% of total sequences) matching 9,433 different known or predicted proteins. The taxonomic classification of hits from the nr database, by species, is represented in Figure 2.

BLASTX search in Swiss-Prot section of the UniProtKB database, identified 11,088 transcripts (20.06%) with significant matches against 7,111 different well-annotated proteins.

### BLASTN against the main nucleotide database

The BLASTN search against the NCBI nucleotide database (nt) identified significant similarity for 10,195 transcripts (18.44%) with 4,509 different subjects. Among sequences with a significant match against nt, 5,366 had not previously been matched against nr and Swiss-Prot databases. Considering all the BLAST searches performed so far, a total of 17,734 ESTs obtained at least one hit, representing 32% of the Adriatic sturgeon transcriptome.

### Evaluation of the unannotated fraction

A total of 43,093 non-redundant transcripts remained unannotated after the BLAST search against the nr database. ORF prediction showed that 41,935 of these sequences (97.31%) contain a putative open reading frame.

### Evolutionary comparison with other fishes

The non-redundant contigs of the two *A. naccarii* libraries were compared to Ensembl release 66 complete cDNA sets for the species of the RS-list.

TBLASTX and BLASTX best hit results are collected in Tables 4 and 5 respectively. The fraction of *A. naccarii* transcripts that identify putatively orthologous genes in other fish and humans reflects the phylogenetic distance between *A. naccarii* and other species. The two non-teleost species on the RS-list, the Sea Lamprey and the Coelacanth, share a higher fraction of genes 33.49% and 30.57% respectively as also confirmed at the protein level (31.97% and 29.24%). A possible explanation for this finding is that these two species separated from the ancestor of teleosts before the Whole Genome Duplication (WGD) known to have interested the teleost clade [15]. Part of the newly-formed genetic material is known to have persisted after duplication, possibly evolving new functions and thus becoming unrecognisable by sturgeon ESTs. This process of gene diversification results in a reduction of the percentage of detectable genes in teleosts. However, a careful analysis of the matching transcripts and proteins should be performed to confirm this hypothesis.

**Table 4:** **TBLASTX best hit (e-value < 1e-03) of *A. naccarii***

	**Lamprey**	**Coelacanth**	**Danio**	**Stickleback**	**Medaka**	**Fugu**	**Tetraodon**	**Human**
reference cDNAs (#)	11,476	21,958	48,636	27,628	24,662	48,003	23,265	180,654
reference genes (#)	10,449	19,174	27,948	20,839	19,687	18,685	19,749	47,266
***A. naccarii ESTs with hit on reference cDNAs (#)***	***7,431***	***12,428***	***13,068***	***11,528***	***11,270***	***11,143***	***10,886***	***12,740***
*A. naccarii* ESTs with hit on reference cDNAs (%)	13.44	22.48	23.64	20.85	20.39	20.16	19.69	23.05
reference genes identified (#)	3,499	5,862	6,396	5,710	5,565	5,447	5,429	6,116
***reference genes identified (%)***	***33.49***	***30.57***	***22.89***	***27.4***	***28.27***	***29.15***	***27.49***	***12.94***

Sequences from known-, novel- and pseudo-gene predictions, from Ensembl realise 66, were collected for the following species: *Petromyzon marinus, Latimeria chalumnae, Danio rerio, Gasterosteus aculeatus, Oryzias latipes, Takifugu rubripes, Tetraodon nigroviridis* and *Homo sapiens*. *A. naccarii* transcriptome sequences were searched against each database. For each sequence, the best hit was annotated.

**Table 5:** **BLASTX best hit (e-value < 1e-03) of *A. naccarii***

	**Lamprey**	**Coelacanth**	**Danio**	**Stickleback**	**Medaka**	**Fugu**	**Tetraodon**	**Human**
reference proteins (#)	11,429	21,817	41,693	27,576	24,661	47,841	23,118	97,041
reference genes (#)	10,402	19,033	26,160	20,787	19,686	18,523	19,602	21,860
***A. naccarii ESTs with hit on reference proteins (#)***	***7,052***	***11,264***	***11,530***	***11,010***	***10,816***	***10,766***	***10,359***	***11,102***
*A. naccarii* ESTs with hit on reference proteins (%)	12.76	20.38	20.86	19.92	19.57	19.47	18.74	20.08
reference genes identified (#)	3,326	5,565	5,865	5,526	5,403	5,346	5,283	5,478
***reference genes identified (%)***	***31.97***	***29.24***	***22.42***	***26.58***	***27.45***	***28.86***	***26.95***	***25.06***

Best hits from the alignment of *A. naccarii* transcriptome sequences against all translations from known-, novel- and pseudo-gene predictions in Ensembl realise 66 for the different species considered in this work.

In any case, the number of putatively orthologous genes matched by *A. naccarii* transcripts in other species is expected to be influenced not only by the genetic similarity among species but also by different parameters such as the accuracy of the genome characterisation in the different species used for the comparison and their evolutionary history in which, for example, different mutation rates may play an important role. In fact, different lines of Osteichthyes are known to have very different evolutionary rates as a result of different factors such as metabolic features or generation times [16]. These differences may deeply affect the number of genes that can be recognized as orthologous among species. Zebrafish seems to share fewer genes (22.89% through transcripts and 22.42% through proteins) with *A. naccarii* than do other teleosts but, the fraction of the *A. naccarii* matching ESTs is comparable to other species. The conclusion is that the Danio genome seems to have a higher number of genes. However this could have a different explanation: first, the number of genes is actually higher according to the high level of genes retention after the WGD hypothesized for this species [17] second, more simply, this result is biased by the more complete genome characterization for this model organism.

### Evaluation of the non-coding RNA component

NcRNA are implicated in every step of gene expression. To discover and annotate potential non-coding RNAs in our transcriptome (miRNA, rRNA, MtrRNA, snoRNA, lncRNA), we searched for genes corresponding to non-coding RNA from genomes of the fish species described above, using BLASTN. Alignment results are collected in Table 6. The highest number of alignments was found against miRNA sequences from the 4 teleosts, in particular in Medaka, whose 9 miRNA were found to be homologous in sturgeon. Mitochondrial and ribosomal RNA were next in abundance. Surprisingly, 11 rRNA pseudogenes from humans were found to be homologous in *A. naccarii.* The alignment method used here can underestimate the number of ncRNA detected as different types of ncRNA have different degrees of sequence conservation between species, with miRNA and snoRNA usually well-conserved while longer-functional ncRNA are not [18]. Moreover, lncRNA elements tend to maintain a consensus secondary structure through compensatory base mutations and, therefore, are difficult to detect by sequence alignments alone [19].

**Table 6:** **BLASTN best hit (e-val < 1e-03) of * A. naccarii *transcriptome against non-coding RNA genes from Ensembl database**

	**Lamprey**	**Coelacanth**	**Danio**	**Stickleback**	**Medaka**	**Fugu**	**Tetraodon**	**Human**
reference ncRNA (#)	2,628	2,918	4,431	1,617	735	703	813	9,399
*A. naccarii* ESTs with hit (#)	4	17	28	29	19	21	31	62
***A. naccarii ESTs with hit (%)***	***0.01***	***0.03***	***0.11***	***0.05***	***0.05***	***0.03***	***0.04***	***0.06***
reference ncRNA identified (#)	4	7	11	11	11	7	13	23
***reference ncRNA identified (%)***	***0.15***	***0.24***	***0.25***	***0.68***	***1.5***	***1***	***1.6***	***0.24***

All non-coding RNA genes and pseudogenes in Ensembl realise 66 for the different species were searched against *A. naccarii* transcriptome*.*

### GO annotation

We started the GO annotation from the BLASTX results against nr. GO terms were retrieved from the association to best-hit for 10,036 (18.15%) of the overall 55,282 *A. naccarii* sequences. Protein domains and motif information were retrieved by InterProScan via Blast2GO and corresponding annotations were merged with already existent GO terms. A total of 29,671 contigs provided significant InterProScan information, with only 3,326 of them resulting in GO annotation. After merging, 6,344 unique GO terms (3,811 for biological process, 758 for cellular component, 1,775 for molecular function), were successfully transferred to 8,784 contigs (16%). As expected, the evidence code distribution shows an over-representation of electronic annotations (IEA), although other non-automatic codes, such as Inferred from Direct Assay (IDA) and inferred by mutant phenotype (IMP), were also well represented (see bar-plot in Additional file 6).

**Figure 3:**
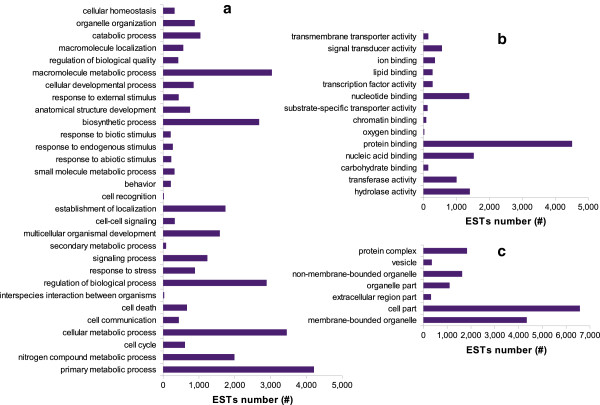
**Distribution of Gene Ontology categories for *A. naccarii *ESTs, across the three domains.***A. naccarii* ESTs were classified into different groups on the basis of generic GO-slim annotations. The bar-plot represents the categories corresponding to level 3 of the DAG graphs built for biological process (**a**), molecular function (**b**) and cellular component (**c**) domains.

*A. naccarii* ESTs were classified by GO-slims within the biological process, molecular function and cellular component domains and a Direct Acyclic Graph (DAG) of the ontologies was generated. Figure 3 shows the number of putative ESTs annotated with high-level GO terms by cutting the DAG graph at level 3 for each of the 3 domains. We also performed enzyme code (EC) annotation through Blast2GO for sequences with GO annotations and retrieved KEGG maps for the metabolic pathways in which they participate. In total 3,634 ESTs were annotated with 448 ECs that identify unique enzymes, participating in 116 different pathways (see Additional file 7). The most populated pathways are “Purine metabolism” (map 00230) with 33 enzymes involved, “Arginine and proline metabolism” (map 00330) with 25 enzymes and Glycolysis/Gluconeogenesis (map 00010) with 22 enzymes.

### Estimation of sequencing completeness

To evaluate the coverage of the cDNA libraries by reads, a rarefaction analysis similar to that described in [20] was employed. The extrapolation from the hyperbolic model, fitted onto the average points, obtained by the 10 replications of sampling and reference transcript identification, using reads from the male library only, showed that 7,293 different transcripts were potentially identifiable in the Danio cDNA set (asymptote “*a*” of the model function). The 6,043 different transcripts actually identified using all reads represents 83% of the theoretical maximum. The angular coefficient calculated at final read count was 0.157. Using reads from the female library only, the number of transcripts actually identified was 5,989, which, compared to the 7,176 maximum transcripts identifiable at infinite sequencing, represents 83% of the total. The slope at the final read count was 0.145. Finally, putting together reads from both libraries, the model-based extrapolation denoted 8,262 different transcripts potentially identifiable, and the 7,286 actually identified represents 88%. The three extrapolated curves are shown in Additional file 8. As expected, the slope at maximum read count was 0.140.

Further analysis, exemplified in Additional file 9, showed that by changing the reference cDNA datasets, the absolute value of the potentially identified transcripts and those actually identified changes, but the ratio between these quantities remains nearly constant. Therefore, the latter ratio is a robust value indicating the fraction of the cDNA libraries really sequenced.

### Estimation of transcriptome completeness

To estimate the total number of A. *naccarii* transcripts potentially present in the two tissues (gonads and brain), we adapted the capture-recapture method widely used in ecology to estimate animal population sizes [21]. This method requires a precise estimate of the fractions of ESTs that can be considered common between the male and female libraries (see method). Since, before joint assembly, each read was labelled with the library of provenance, final contigs were classified according to the origin of their reads as being cDNA3-specific, cDNA4-specific or common [22]. First, we separated 17,399 cDNA3-specific contigs from the male library (31% of the total) and 17,523 cDNA4-specific contigs from the female library (32% of the total). The direct subtraction between the two groups of library-specific contigs isolated 394 contigs showing mutual alignments from each fraction. The indirect subtraction identified 41 cDNA3-specific and 38 cDNA4-specific contigs, that aligned on 85 common subjects. Finally, using NCBI nr as the common database, we identified an additional 13 cDNA3-specific and 12 cDNA4-specific contigs which map onto the same 10 protein sequences. After all subtractions, 16,951 cDNA3-specific and 17,079 cDNA4-specific contigs remained, that may represent potentially sex-distinctive transcripts.

With the Rcapture R package we estimated the transcripts population size to be 68,904 with a standard error of 210. This means that we have probably sequenced about 80% of the total transcripts in the two tissues of *A. naccarii*. Additional redundancy could still be present in the common contig fraction (despite attempts to reduce it in the assembly phase), which would have increased the estimated total number of transcripts in the tissues of origin. By adopting the capture-recapture approach we are aware that the resulting percentages probably represent an over-estimate of the real fraction of captured transcripts. In fact, the procedures used for the identification of the transcripts common to both libraries may cause some bias due, for example, to a non-correct identification of gene families or to different variants of duplicated genes, especially when dealing with a tetraploid species like *A. naccarii*. Moreover, the assumption that the two fish share the same transcriptomes might be bold because the different genders of the two animals could be responsible not only for different genes directly involved in sex determination but also, for example, for differences in the developmental rate. Nevertheless, we think that the approach here proposed, even if indicative when applied to a single comparison, might be very useful for comparative analyses of multiple libraries with the purpose of estimating the relative completeness, especially if obtained by the same sample.

The transcriptome completeness was also evaluated, as noted above, by searching for constitutively-expressed mitochondrial genes. Of the 12 polypeptide coding genes of the white sturgeon *Acipenser transmontanus* mitochondrial genome, 11 were found in our assembly. Only the gene for ATPase subunit 8 was missing. Contigs that aligned with these genes showed between 93 and 100% identity.

### Search for sex-determining genes

We evaluated the presence of 32 candidate genes known to be involved in sex determination and sexual development in vertebrates by queering the transcriptome with 3 collections of orthologs and paralogs for those genes (Ensembl Compara, Homologene, Acipenser-specific genes, see methods). The first collection represents the largest variety of annotated homologous (orthologs and paralogs), from sequenced genomes, categorised in Ensembl Compara. The second collection is represented by clusters of more specific orthologs, downloaded from NCBI HomoloGene [23]. The third group of sequences is a collection of complete or partial CDSs from other sturgeon species of the genus Acipenser available in NCBI GenBank. The use of large collections of putative orthologs and paralogs maximizes the possibility of detecting homologues. In contrast, restricted collections of reliable homologues allow a higher confidence on the match they find. If a contig is confirmed as the best subject for a given gene in searches of all trees, then we can be more confident about its identity.

Significant matches were found for 22 of the 32 genes investigated. The alignments of matching contigs were manually inspected to exclude false-positive matches exclusively due to the presence of widespread protein domains. A complete list of the best matching contigs considered to have a reliable similarity against the 22 genes recognised is contained in Additional file 10. A similar transcriptomic screening for genes involved in sex differentiation was performed on the lake sturgeon (*A. fulvescens*) [24]. The authors report positive matches for 12 genes (SOX2, SOX4, SOX17, SOX21, SOX9, DMRT1, RSPO1, WT1, WNT4, FOXL2, TRA-1, FEM1), all but one included in our search list, the exception being TRA-1. All genes were also detected in *A. naccarii* with the exceptions of DMRT1 and WNT4. Positive matches with SOX genes (SOX2, SOX4, SOX21) were discarded after manual inspection, because the same contigs also matched other SOX genes with higher scores. This multiple matching is due to the fact that genes of the SOX family often share the conserved High Mobility Group box domain and assignment based on this domain makes for a less-reliable identification.

The absence of the DMRT1 gene from both the *A. naccarii* libraries is especially interesting and might be due to the incomplete coverage. A second possibility is that this gene is not expressed at the stage at which our samples were collected. In fact, the animals analysed for this project were six months old and were at an early stage of gonad differentiation. This is, to our knowledge, the first stage at which sturgeons, which cannot be sexed visually, have unambiguous evidence of gonad differentiation through fine histological investigation [25]. The lake sturgeons analysed by Hale and colleagues [24] were estimated to be 13 or 14 years old. All characterisations of DMRT1 genes from other sturgeon species have been performed on mature or sub-mature animals [24,26]. Finally, a low expression of this gene is displayed in the Siberian sturgeon with no evident gonad differentiation [27]. Thus, the absence of DMRT1 in the transcriptome of the very young *A. naccarii* analysed would suggest that this gene is expressed at a later stage of development in this species (and probably in all sturgeons). DMRT1 in known to play an important role as an activator of the genetic cascade of sex differentiation in some other fish, such as Medaka [28]. Even if most of the genes involved in sex determination are known to act in a dosage-dependent manner [29], under the hypothesis that sex differentiation in sturgeon is genetically determined, one could expect that, at the origin of the genetic cascades leading to the different genders, a sex-linked genomic polymorphism occurs. For this reason, special attention was given to the contigs observed to be library-specific. Among the 22 genes detected, only 5 (WT1, LHX1, CYP19A1 (aromatase), FHL3, FEM1A) and 2 (AR, EMX2), were found to be specific to male (cDNA3) or female (cDNA4) libraries. These genes represent, in our opinion, interesting candidate transcripts for experimental validation by PCR amplification. The remaining fifteen genes were detected by contigs belonging to the common fraction.

### Discovery of variants

At 90% Bayesian probability, we were able to identify 23,084 SNPs and 59,150 INDELs. After having filtered out variants beside simple sequence repeats, 21,791 SNPs (94.04%) and 57,996 INDELs (98.05%) were retained from 6,283 and 8,678 contigs respectively. Between contig-containing variants, the average SNP per contig was 3.5, while the mean INDELs per contig was 6.7. The mean frequency across all contigs was 1 SNP every 1. Kbp, and 1 every 377 bp for the INDELs. We identified 14,433 transitions (Ts) and 7,358 transversion (Tv), thus confirming that transitions are more common than transversions in our dataset [30].

**Figure 4:**
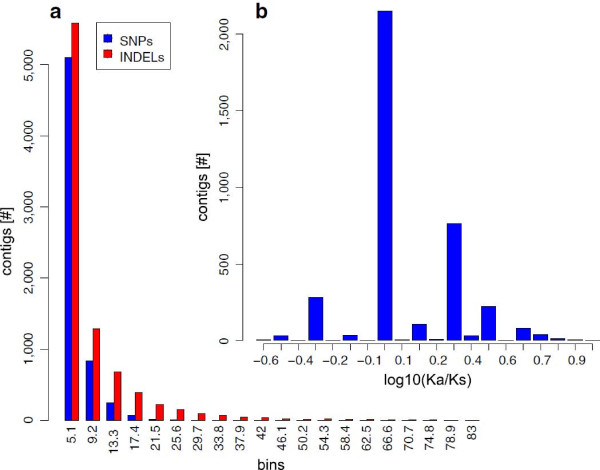
**Distribution of SNPs and INDELs across *A. naccarii *contigs. a**) Bar-plot of the distribution of SNPs and INDELs in contig-containing variants. Most contigs contain up to 5 variants. **b**) Bar-plot of log10 distribution of Ka/Ks for contig-containing SNPs that lie in predicted ORFs. Contigs with Ka/Ks > 1 (log10 > 0) are proposed to be under diversifying selection in *A. naccarii.*

**Figure 5:**
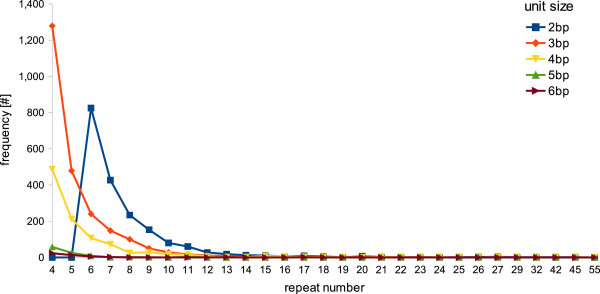
**Frequency of classified SSR repeat types in *A. naccarii.*** The graph shows the frequency of each repeat motif classified, considering the sum of the frequencies for complementary sequences (for example, the sum of frequencies for the dinucleotides AC and its complementary GT), for the 5,295 total SSRs identified in 4,670 contigs.

We then classified SNPs that fell in predicted coding regions according to the type of mutations: non-synonymous (Ka) or synonymous mutations (Ks). Of the overall contig-containing SNPs, we were able to identify a putative ORF for 2,482 of them on the basis of the best match against nr database, while for 3,786 an ORF was predicted. Of the overall SNPs found in coding regions, 2,750 represented non-synonymous mutations while 1,056 were synonymous. We found that 1,280 contigs (2.32% of all contigs) had Ka/Ks > 1 thus indicating genes putatively under diversifying selection within our samples. On average, we found 0.73 non-synonymous and 0.28 synonymous SNPs per contig in coding regions; this means one non-synonymous mutation every 9 Kbp of coding portion, and 1 synonymous mutation every 20.7 Kbp. Distribution of SNPs and INDELs across contigs together with distributions of Ka/Ks are shown in Figure 4. We also scanned the entire EST set for Sample Sequence Repeats (SSRs, also known as microsatellites), and we identified 5,295 SSRs present in simple formation, within a total of 4,670 (8%) contigs. In particular, we found 1,891 dinucleotides, 2,377 trinucleotides, 1,001 tetranucleotides, 100 pentanucleotides and 45 hexanucleotides. The graph in Figure 5 shows the frequency of repeat types found accordingly to unit size. Of the overall contig-containing SSRs, 4,639 also contain a putative ORF. In total 1,779 SSRs are predicted within ORFs (33% of all identified SSRs). This availability of a relevant number of EST-linked microsatellites and SNPs represents a precious prerequisite for sturgeon conservation genetics by providing the possibility to monitor the effect of selection on captive and released stocks.

### AnaccariiBase: a free genomic resource for *A. naccarii*

Freely available at: http://compgen.bio.unipd.it/anaccariibase/, AnaccariiBase contains *A. naccarii* transcriptome information and results of bioinformatics analysis, organised in different layers. The database is focused on contig sequences and annotations, and can be searched through contig ID and key-words. Moreover, it allows the user to conduct a local BLAST search on the fly against contigs to identify one or more transcript significantly similar to a given query sequence. Furthermore the system provides a customizable data retrieval tool to download large amounts of data. The information layers are detailed hereafter. (1) Contig information. For each contig, an ID is given together with the FASTA sequence and an informative description, which is defined by the Blast2GO natural language text mining functionality, related to the BLAST hits. The best hit is used when a Blast2GO description is unavailable. (2) Assembly. The list of the reads assembled into each contig is accessible to the user, together with their sequences. (3) Gene Ontology. GO terms associated to each transcript are given for Biological Process, Molecular Function, and Cellular Component domains, with hyper-link to the GO database. (4) BLAST results. Pre-calculated BLAST results of contigs against the main nucleotide and protein databases, are shown in the classic BLAST output format. Results are hyperlinked to the external databases, and include alignment descriptions and details about the pairwise alignments of each contig with the corresponding BLAST hits.

## Conclusions

The present study provides the first insight into the transcriptome of the Adriatic sturgeon, a critically endangered species endemic to the Adriatic Sea. More generally, this is also the first large release of transcriptomic information for a sturgeon species, shared through a dedicated and searchable database. With over 55,000 high quality sequences, the information reported represents a significant advance in sturgeon genetics. The apparently limited fraction of successfully annotated sequences with GO terms (16%) might be due to the very ancient separation (about 250 MYBP) of sturgeons from any other species for which a relevant genomic information is available. Additionally, following the sturgeon-specific Whole Genome Duplications [4] part of the redundant genetic information probably underwent a functional divergence that may have further decreased the fraction of successfully annotable ESTs. Beside the evolutionary interest of a database obtained from a member of the Chondrostean, certainly applied genetics studies on sturgeons will benefit from this resource. The present paper also report the results of an investigation on genes related to sex differentiation. Out of the 32 genes investigated 7 were detected in only one of the two libraries suggesting a possible differential expression between genders at this early stage of gonad differentiation. This result might be affected by the limited coverage of our sequencing and should be considered as a starting point for further investigations. Interestingly, DMRT1, a master gene for the sex determination known to be expressed in both sexes in different sturgeon species was not detected, suggesting that, differently from other fish species, DMRT1 is expressed in sturgeons only in latter stages of maturity. Finally, the availability of thousands of EST-linked microsatellites makes possible the establishment of a genome-wide genetic markers panel useful to monitor the effect of different selective pressures and to monitor the effects of restocking practices. Restocking of most sturgeon species depends on *ex situ* conservation because of the dramatic decline of natural populations [1]. In synthesis, the data provided in the present study and shared through a dedicated website represents the first substantial release of information on a sturgeon transcriptome and will hopefully constitute a useful contribution to sturgeon genetics, aquaculture, and conservation.

## Methods

### Preparation of samples, construction of cDNA libraries and sequencing

Two 6-month-old individuals were collected from the “Azienda Agricola VIP” farm (Orzinuovi, Brescia, Italy). Their sex was determined by histological analysis of the gonads as being one male and one female. Animals were anaesthetised with chloretone and painlessly killed. No analyses or experiments were conducted with live animals. The University of Padova ethic board CEASA (Comitato Etico di Ateneo per la Sperimentazione Animale) exempted this study from review as an extra moenia activity. Biopsies were performed from gonads and brain for RNA purification and a part of the gonads was used for sex determination according to the procedures described in Grandi and Chicca [25]. An RNeasy mini-column kit (QIAGEN) was used to extract total RNA from 30 mg of each tissue from each individual. Total RNA was checked for integrity, purity and size distribution. RNA samples from each individual were pooled and stored in three volumes of 96% ethanol and 0.1 volume of sodium acetate to obtain 5 μg of pooled RNA in a final volume of 120 μl. Pooled RNA was sent to Evrogen (Moscow, Russia; http://www.evrogen.com). The SMART (Switching Mechanism At 5′ end of RNA Template) kit was used to retrotranscribe total polyadenilated RNA. First-strand cDNA synthesis was performed with SMART Oligo II oligonucleotide (5`-AAGCAGTGGTATCAACGCAGAGTACGCrGrGrG-3′) and CDS-GSU primer (5′-AAGCAGTGGTATCAACGCAGAGTACCTGGAG-d(T)20-VN-3′) using 0.3 μg of total RNA. Double-strand cDNA was obtained from 1 μl of the first-strand reaction (diluted 5 times with TE buffer) by PCR with SMART PCR primer (5′-AAGCAGTGGTATCAACGCAGAGT-3′). Amplified cDNA PCR product was purified using QIAquick PCR purification Kit (QIAGEN, CA). The two SMART prepared libraries were then normalised using the duplex-specific nuclease (DSN) method [31]. Normalisation included PCR amplification of the normalised fraction.

In order to gain more material, 30 ng of normalised cDNA were used for 100 μl PCR and 7 cycles of PCR amplification with SMART PCR primer were performed as suggested by Evrogen (http://www.evrogen.com/kit-user-manuals/Trimmer-2.pdf). Moreover, in order to decrease the possibility to introduce biases due to PCR, the above amplification was independently replicated 20 times and the products pooled before sequencing. Adapters were trimmed using GsuI (Fermentas) following the standard protocol and cDNA purification was performed with Agencourt AMPure XP (BECKMAN COULTER). BMR Genomics, University of Padua, Italy (http://www.bmr-genomics.it), prepared and sequenced 454 protocol libraries. Approximately 15 μg of normalized cDNA from each library were sequenced in a 1/4 picotiter plate on a Genome Sequencer FLX instrument using GS FLX Titanium series reagents.

### Cleaning and assembly

The raw reads from every library were extracted from 454 SFF files through the open source alternative sff_extract 0.2.10. Summary control of raw reads' quality was done with FastQC 0.10.0. Sequences were cleaned using the est_process module driven by preprocessest.pl into the est2assembly 1.13 package [32] that perform sequencing adaptor removal, low complexity region masking, quality trimming, and poly A/T detection and removal. After preprocessing, *A. naccarii* male and female reads were tagged accordingly to the library of origin and jointly assembled, thus allowing contigs to be classified for reads content as being composed by males only, by females only or by both sexes. Sequences of the two libraries were jointly assembled by MIRA 3.2.1 [33]. The obtained contigs and singletons were further re-assembled by performing a second round to decrease the redundancy caused by the heuristic nature of the assembly process [34]. In the first run (*de novo* assembly), all cleaned reads were used as input and processed with the following parameters: -job = denovo, est, accurate, 454; -LR:fo = no, -SB:lsd = no, -CL:cpat = 0:qc = 0, -ED:ace = 1, -OUT:sssip = yes, -CO:fnicpst = yes, -LR:mxti = no, -AS:mrpc = 1. In the second run the following parameters were used: -job = denovo, est, accurate, 454, -notraceinfo, -LR:fo = no, -CL:cpat = 0:qc = 0, -ED:ace = 1, -OUT:sssip = yes, -CO:fnicpst = yes, -LR:mxti = no, -AS:mrpc = 1. After the second assembly step, the native reads of each contig (from the first round) or metacontig were traced back through SSAHA2 2.5.4 [35] with parameters (−rtype 454 -output sam). For each of the above contigs or metacontigs, the alignment of the corresponding reads resulted in a SAM file allowing the calculation of the coverage using SAMtools [36] and BEDtools [37].

### Functional annotations

BLASTX 2.2.25+ [38] similarity searches for the entire transcriptome were conducted locally against the NCBI non-redundant (nr) database (downloaded 2010/10/19) as the first *de novo* annotation step. The Swiss-Prot part of the UniProt database (downloaded on 2012/02/24) was also queried. Local TBLASTX 2.2.25+ similarity searches were conducted locally against (1) 6,088 EST sequences of the genus *Acipenser* downloaded from NCBI-taxonomy (2011/04/18), mainly obtained from *A. sinensis*[39] and *A. transmontanus*[40] (2) a super-set of all transcripts resulting from Ensembl (release-66) including known-, novel- and pseudo-gene predictions for the following list of reference species (hereafter RS-list): *Petromyzon marinus, Latimeria chalumnae, Danio rerio, Gasterosteus aculeatus, Oryzias latipes, Takifugu rubripes, Tetraodon nigroviridis* and *Homo sapiens.* In order to identify non-coding sequences, BLASTN 2.2.25+ similarity searches were conducted locally against whole non-coding RNA gene and pseudogene sequences from Ensembl release-66 for the species of the RS-list. A BLASTN similarity search was also performed against the NCBI nucleotide sequence (nt) database (downloaded on 2012/02/24). BLASTX, BLASTN and TBLASTX searches were carried out using default parameters.

Given the high evolutionary distance among the species compared, alignments with an e-value < 1e-03 were considered significant and a maximum of 20 hits were taken into account for each query. The taxonomic classification of annotations was performed by MEGAN 4 [41] based on the absolute best BLAST hits. Contigs with multiple best BLAST hits were excluded from the count. The mapping of GO annotations to contigs was achieved with Blast2GO 2.4.7 [42]. Annotations were conducted only for contigs with significant BLASTX hits below e-value 1e-06, with 55 as the annotation cut-off and 5 as the GO weight. No HSP-hit coverage cut-off was used. InterProScan annotation was also conducted via Blast2GO. Obtained information for domains was included to improve global annotations.

### Estimation of sequencing completeness

To test how completely our physical cDNA libraries were sequenced, we adopted the method described in Franssen et al. [20], based on saturation curve calculation. From the total cleaned reads pool, increasing subsets of reads were randomly selected and, for each read, the corresponding contig in which it was assembled was traced back. Detected contigs were blasted against a reference cDNA set using TBLASTX with the e-value cut-off at 1e-03. The best matching subject was recorded for each contig. The sampling was repeated 20 times with a constant increase in sample size, reaching the totality of cleaned reads in the last run, thus identifying, in the end, 20 pools of different reference cDNAs. The number of matching reference cDNAs at each cycle was plotted against the corresponding reads sample size and a hyperbolic model *y* = *ax*/(*b* + *x*) was fitted to the points by non-linear regression to assess the parameters “*a*” and “*b*” with “*a*” representing the upper limit of the model function, i.e., the maximum theoretical number of reference transcripts identifiable by the initial cDNA libraries if these had been exhaustively sequenced. Moreover, the slope of the hyperbolic curve at maximum sample size gives an evaluation of how quickly the asymptotes “*a*” will be reached, thus indicating the decreasing potential to detect additional transcripts. We built saturation curves by sampling cleaned reads from: 1) male only, 2) female only and 3) joint libraries. In all cases, we mapped reads back to the final assembly contigs. The whole cDNA super-set from *Danio rerio* in Ensembl release-66 was chosen as the reference. However, our analysis demonstrated that the fraction of detected reference transcripts, with respect to the maximum estimated, and the slope of the curve at maximum sample size do not substantially change using different cDNA sets as a reference (see Additional file 9).

### Estimation of transcriptome completeness

We inferred the total transcripts population size in the two *A. naccarii* samples by estimating the number of transcripts shared by the two independent libraries taking into account that the two animals analysed had the same age and the same history. By neglecting the differences due to sex-specific transcripts, the two sequence libraries were handled as two sampling replicates from the same transcripts' population. The fraction of the transcript from the first library that is also represented in the second one is a direct estimate of the completeness of the second library and vice-versa. The same approach has already been applied to estimate the number of human genes [43].

Since each read was labelled with the library of origin before joint assembly, final contigs were classified as being “male_library-specific”, “female_library-specific” or “common”. The common one is the fraction of contigs composed of reads of both libraries and then represented by transcripts considered to be shared by the two libraries. We performed a direct subtraction, i.e. a bidirectional BLASTN, between the libraries to identify the contigs that were not library-specific. Library-specific contigs that align for more than 80% of their length, with e-values below 1e-50 were moved into the common fraction. We also performed an indirect subtraction to take into account contigs representing partially-overlapping or non-overlapping portions of the same long transcript, which had not been assembled together due to the lack of a sufficient link. Both groups of library-specific contigs were searched for similarities, using TBLASTX, against cDNA sets resulting from Ensembl release-66 for the RS-list species. All cDNA sequences provided by NCBI-Taxonomy Browser for the genus Acipenser (2011-04-18) were also screened. Protein sequences available for other species were assessed by searching the NCBI nr database (2010/11/02) with BLASTX. The library-specific contigs matching the same subjects, with 1e-06 as the e-value threshold and > 80% query coverage were moved into the common fraction. We exploited the Rcapture R package [44] to estimate the total transcripts population sizes because it allows the association of a standard error to the obtained estimation. Furthermore, we assessed the completeness of the *A. naccarii* transcriptome by screening for the presence of the 13 polypeptide coding genes [45,46] from the complete mitochondrial genome (mt) of *Acipenser transmontanus* (GenBank accession no.: AB042837) using BLASTN with a 1e-10 e-value threshold.

### Search for sex-determining genes

We obtained sequences from genes known to be involved in sex determination and sexual development in vertebrates from different species and used them to search our assembled contigs by similarity in order to investigate the content of library-specific contigs, isolated by *in silico* subtraction in more detail. The genes and gene families considered were: WT1, LHX1, CYP19A1, FHL3, FEM1A, AR, EMX2, DAX1, SOX9, SOX17, SOX1, SOX11, SOX6, SOX14, FOXL2, RSPO, SF1, FGFR2, FGF9, GATA4, LHX9, ATRX, SOX2, SOX4, SOX21, WNT4, SRY, STRA8, FIGLA, AMH, VTG2, DMRT1 [47-49]. We obtained sequences in 3 different ways: 1) Ensembl database annotated orthologous and paralogous of the above genes were identified, starting from the well-annotated Zebrafish genome in Ensembl 66, by querying each common name. For each gene, we identified all orthologous and paralogous within Ensembl Compara version 66. Then, for each ortholog and paralog, all alternative transcripts were identified and the corresponding protein sequence downloaded. 2) Clusters of homologs (paralogs and orthologs) of candidate genes were identified within NCBI HomoloGene Release 66 and corresponding protein sequences were downloaded. 3) Nucleotide sequences for genes FOXL2, DMRT1, and SOX used as references in a previous scientific study aimed at gender identification in the Shovelnose sturgeon (*Scaphirhynchus platorynchus*) [26] together with corresponding sequences from other sturgeons of the genus Acipenser were downloaded from NCBI Genbank (15/10/2012). Each group of paralog and ortholog protein and nucleotide variant representing a gene was searched for similarity in our transcriptome assembly using TBLASTN and BLASTN respectively. Alignments with an e-value > 1e-03 and fewer than 50 positive matching nucleotide/aminoacid positions in the BLAST alignment were discarded. Each different contig that presented a match was extracted for each gene. For each contig (subject) matched by more than one homologue (query), the homologue with the highest alignment bit score was selected. Results obtained by the three approaches were compared for each gene and the more-likely contig was selected based on the following criteria: 1) BLAST alignment bit-score with the query; 2) per-base mean coverage (singletons were discarded); 3) nucleotide alignments between candidates to ensure they actually represented distinct sequences (using MAFFT v6.935b [50]); 4) alignments between contig translations and corresponding protein queries (using MAFFT); 5) presence of one or more distinctive and important functional domains encoded by the target gene within the translated and aligned fraction of contigs (by searching in Pfam-A version 26.0 [51]); 6) the ratio between the length of the translated-aligned fraction and the total contig length; 7) consistency of annotations obtained by blast2GO via alignment against all protein sequences included in the NCBI non-redundant database.

### Discovery of variants

Since mean contig coverage is generally low (< 5X) and the transcriptome comes from different individuals, we adopted a method based on a probabilistic framework, which allows the estimation of uncertainty regarding variants calling, in order to identify SNPs and short INDELs [52]. We used Freebayes 0.9.4 [53] which employs Bayesian formulation to calculate the probability that multiple different alleles are present between the reference and the aligned reads. Freebayes is also able to call variants from polyploid pooled samples. SAM alignments calculated for each contig in the assembly phase were input into Freebayes with the following parameters: probability cut-off of 0.9, 5 as the minimum coverage required to process a site, and each SNP must be supported by at least 2 reads. As it has been shown that improved base-call accuracy can lead to a significant reduction in false-positive SNP calls, base alignment quality (BAQ) adjustment was applied to the input alignments through SAMTOOLS calmd 0.1.18 [36]. Homogenisation of the potential insertion and deletion distribution through reads-independent left realignment to improve the INDELs call was obtained by the bamleftalign tool included in the Freebayes package. It is known that variant calling near repetitive DNA sequences are prone to error, especially in 454 technology where over-calls or under-calls of repetitive stretch, are the most common errors [54]. We then filtered out all variants that were beside 4 repetitions of any sample sequence repeats (including homopolymeric regions). For each contig containing SNP, we calculated the number of transitions (Ts) and transversions (Tv). The mutation resulting from each SNP was characterized in terms of synonymous (Ks) or non-synonymous (Ka), and location (inside the ORF or in the 5′ or 3′ UTR regions). For each contig-containing SNP with a BLAST hit against the nr database, the ORF was deduced from the alignment against the best HSP. For contig-containing SNPs without a hit, the ORFs predicted by the ORFpredictor were used. We then calculated the ratio (Ka + 1)/(Ks + 1) where, 1 was added to enable the calculation of the ratio even when Ks = 0. To find both perfect and imperfect microsatellite repeats (SSRs) for di-, tri-, tetra-, penta- and hexa-nucleotides in unit-size, within our contig sequences, we adopted the MISA tool version 1.0 [55], with min_repeat specifications of 6, 4, 4, 4, and 4 respectively. These thresholds are in agreement with the minimum lengths recommended for repetitions outlined in [56], to allow the polymerase slippage events, which makes the identified microsatellites, potentially polymorphic. We set at 0 the maximal number of nucleotides that interrupt compound microsatellites. Moreover we distinguished SSRs within ORFs predicted by BLAST comparisons or in alternative by ORFpredictor.

## Competing interests

The authors declare that they have no competing interests.

## Authors’ contributions

MV conceived the study, performed the bioinformatics analysis and wrote the manuscript. AG: participated to the study design and to the preparation of the manuscript. EB and FB performed the laboratory parts of the study. AC: built the web-database. GG: performed the sex determination by histological analysis of the gonads AK: participated in the design of the study. LC: conceived and coordinated the study and wrote the manuscript. All authors read and approved the final paper.

## Supplementary Material

Additional file 1 **Distribution of cleaned-read lengths for the *A. naccarii *male (cDNA3), female (cDNA4) and the joined libraries.** Bin intervals are shown along the x-axis. Click here for file

Additional file 2 **Distribution of contig- and singleton- lengths for *A. naccarii *first round and final assemblies.** While the average quality of singletons remains between 15 and 40, the average quality of assembled contigs rises to 88. Click here for file

Additional file 3 **Distribution of contigs' and singletons' average quality for first round and final assemblies.** The figure shows how the number of singletons and contigs resulting from the first assembly (largest contig 2,732, N50 contig size 489, N90 contig size 324, N95 contig size 258), is reduced in the final set. Click here for file

Additional file 4 **Mean contigs coverage distribution for the first and final assemblies.** Percentage of contigs falling in the different coverage intervals are referred to the final assembly. The average coverage of the contigs is quite low. As shown on the graph where about 61% of contigs have average coverage of only up to 3 per base. Click here for file

Additional file 5 **Pair-wise relationships between main properties characterising total contigs obtained by the first and final assemblies.** Pair-wise relationships between lengths and qualities (A, D), lengths and number of reads per contig (B, E), qualities and mean reads per contig (C, F), in the set of 60,825 contigs from the first assembly and 55,282 contigs from the (second) reassembly of the *A. naccarii* transcriptome. Click here for file

Additional file 6 **Evidence code distribution of the annotation of *A. naccarii *transcriptome.** Only the evidence codes assigned to at least one sequence are reported. EXP: Inferred from Experiment, IDA: Inferred from Direct Assay, IPI: Inferred from Physical Interaction, IMP: Inferred from Mutant Phenotype, IGI: Inferred from Genetic Interaction, IEP: Inferred from Expression, Pattern, ISS: Inferred from Sequence or Structural Similarity, ISO: Inferred from Sequence Orthology, ISA: Inferred from Sequence Alignment, ISM: Inferred from Sequence Model, RCA: inferred from Reviewed Computational Analysis, TAS: Traceable Author Statement, NAS: Non-traceable Author Statement, IC: Inferred by Curator, ND: No biological Data available. Click here for file

Additional file 7 **KEGG pathways found in the *A. naccarii *transcriptome.** Enzyme Codes were mapped on the sequences with GO annotations through Blast2GO, then metabolic pathway map numbers in which enzymes carry out their function were retrieved, thus identifying 833 different enzymes participating in 116 different pathways. Click here for file

Additional file 8 **Saturation curve for male, female and both *A. naccarii *cDNA libraries.** Read subsets of increasing sample size were randomly extracted from total pool in the library. For each subset, contigs in which reads where assembled were identified. Each contigs pool was used to identify *Danio rerio* cDNAs (TBLASTX 2.2.25+ e-value 1e-03). Re-sampling and identification process was repeated 10 times for each sample size. A mean value and a confidence interval for the number of identified *Danio* cDNAs was calculated for each sample size. Hyperbolic model *y = (ax)/(b + x)* was fitted on points given by sample size versus average cDNAs hit so that model parameters “*a*” and “*b*” were estimated. The legend shows estimated parameter values obtained by fitting the hyperbolic model on the data. As can be seen, the curves from the single libraries retain the same trend and the difference is mostly due to the different number of reads in each library. Click here for file

Additional file 9 **Saturation curves plot for joint *A. naccarii *cDNA libraries against cDNA sets from other fishes.** Were constructed several saturation curves, starting from total reads from the two libraries and using different sets of cDNA as a references. cDNA sets used are derived from all transcripts from Ensembl 66 for species in RS-list. The estimated parameters of the curves are reported in the legend. Click here for file

Additional file 10 **Sex related genes found in the Adriatic sturgeon transcriptome.** The table lists the contigs of the *A. naccarii* transcriptome that best represent 22 of the 32 genes known to be involved in sex determination and sexual development of vertebrates, used for the screening. For each recognized gene are shown: the gene symbol; the gene extended name; the queries from the different sources that gave the best alignments with the defined thresholds, the NCBI HomoloGene cluster ID the query belongs to (for queries from this source); the Ensembl ID (for queries from this database), the GenBank Accession (possibly for all queries); the contig that best represents the putative *A. naccarii* orthologous (subject); the assembly fraction the contig belongs to (cDNA3, cDNA4, or common); its mean per-base coverage; the bit score of the alignment between the contig and its query; the contig translated-aligned fractions on the query; the putative Pfam domains contained within the translated and aligned fractions; finally the contig annotation given by Blast2GO. Click here for file
