# Supplementary figures and images for: Transcriptome sequencing and de novo annotation of the critically endangered Adriatic sturgeon
Source: BMC Genomics. 2013 Jun 18;14:407. doi: 10.1186/1471-2164-14-407 (PMC3691660; doi:10.1186/1471-2164-14-407)

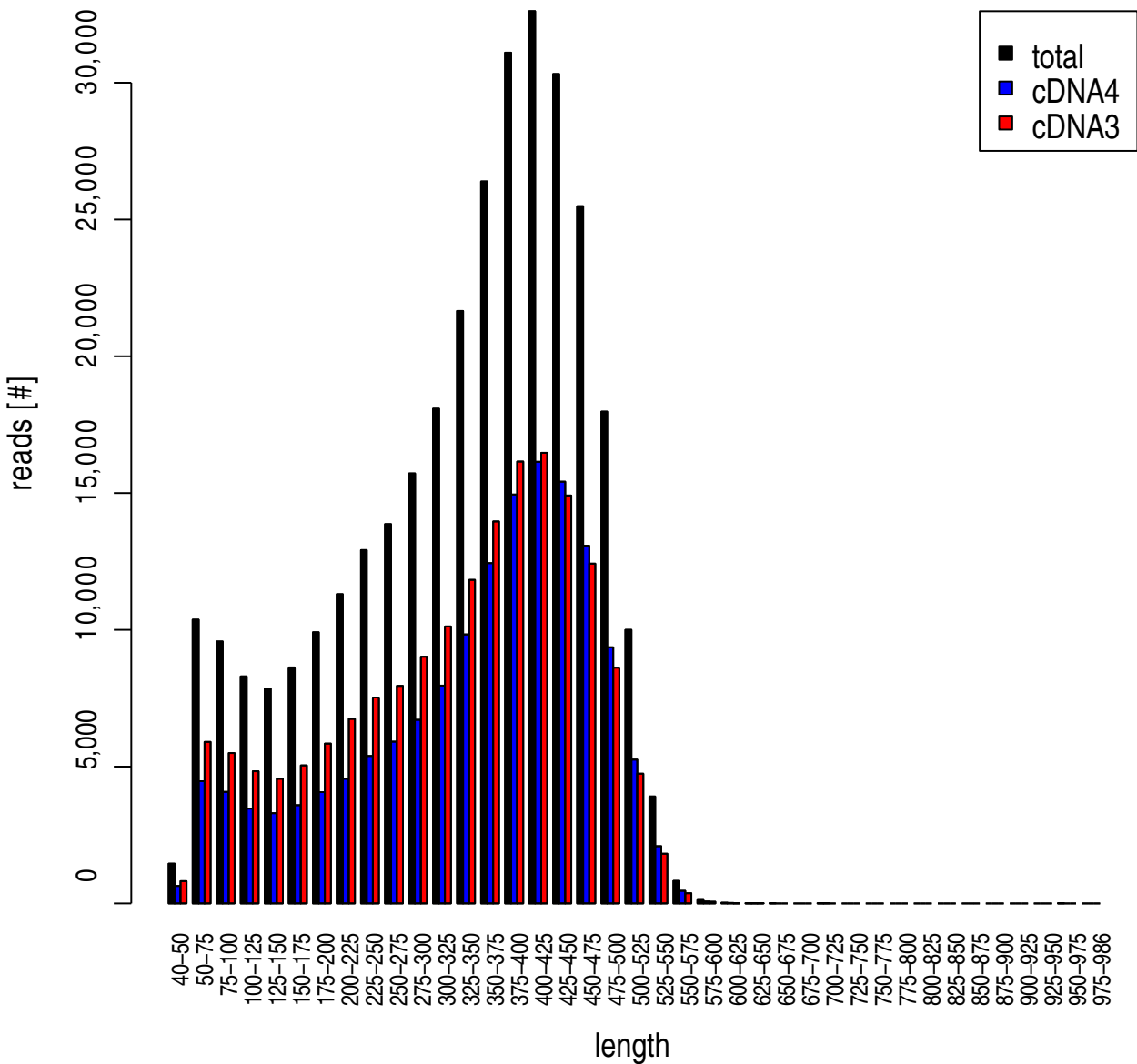

Supplement: Additional file 1 — Distribution of cleaned-read lengths for the A. naccarii male (cDNA3), female (cDNA4) and the joined libraries. Bin intervals are shown along the x-axis. [file 1471-2164-14-407-S1.pdf]

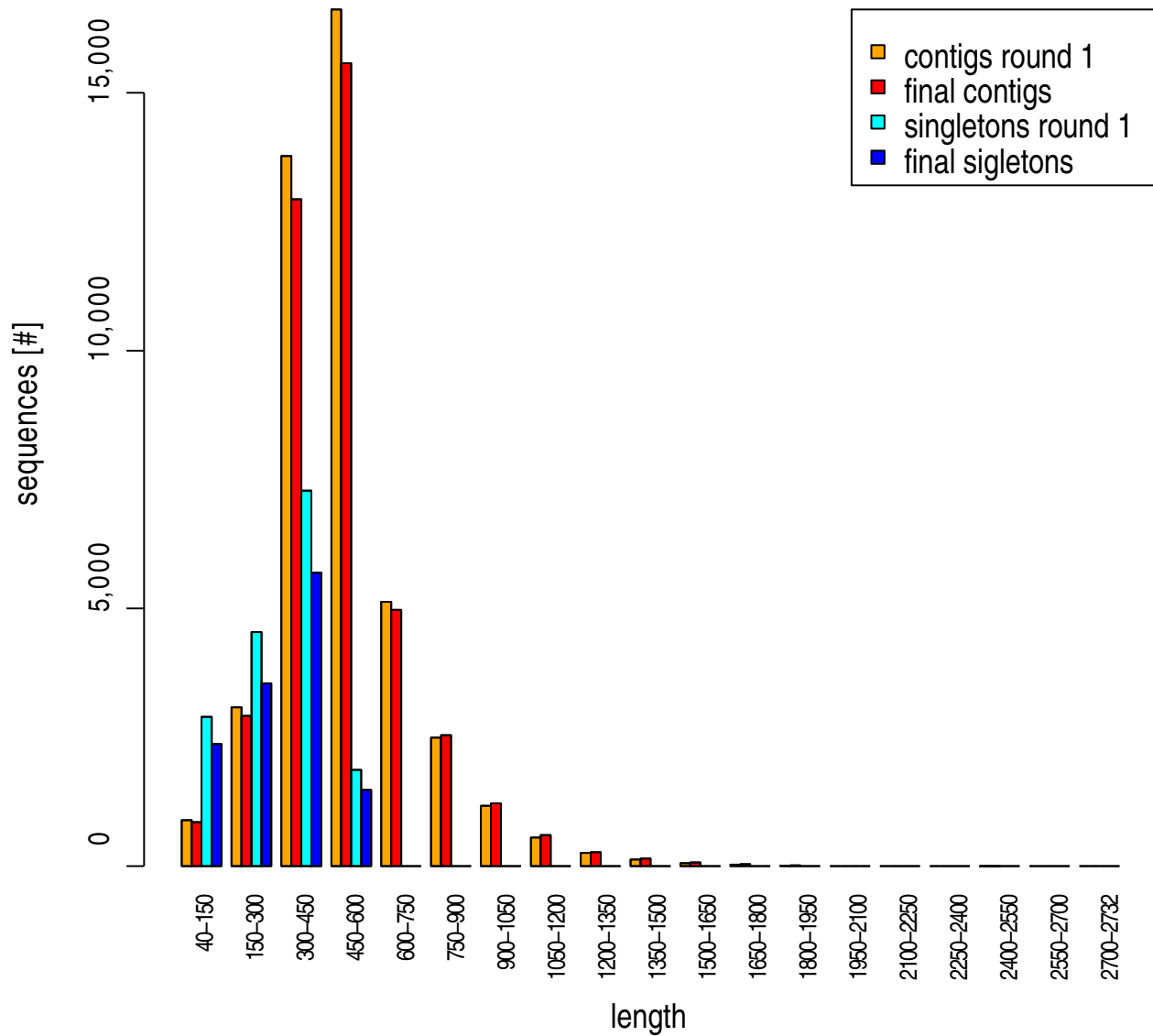

Supplement: Additional file 2 — Distribution of contig- and singleton- lengths for A. naccarii first round and final assemblies. While the average quality of singletons remains between 15 and 40, the average quality of assembled contigs rises to 88. [file 1471-2164-14-407-S2.pdf]

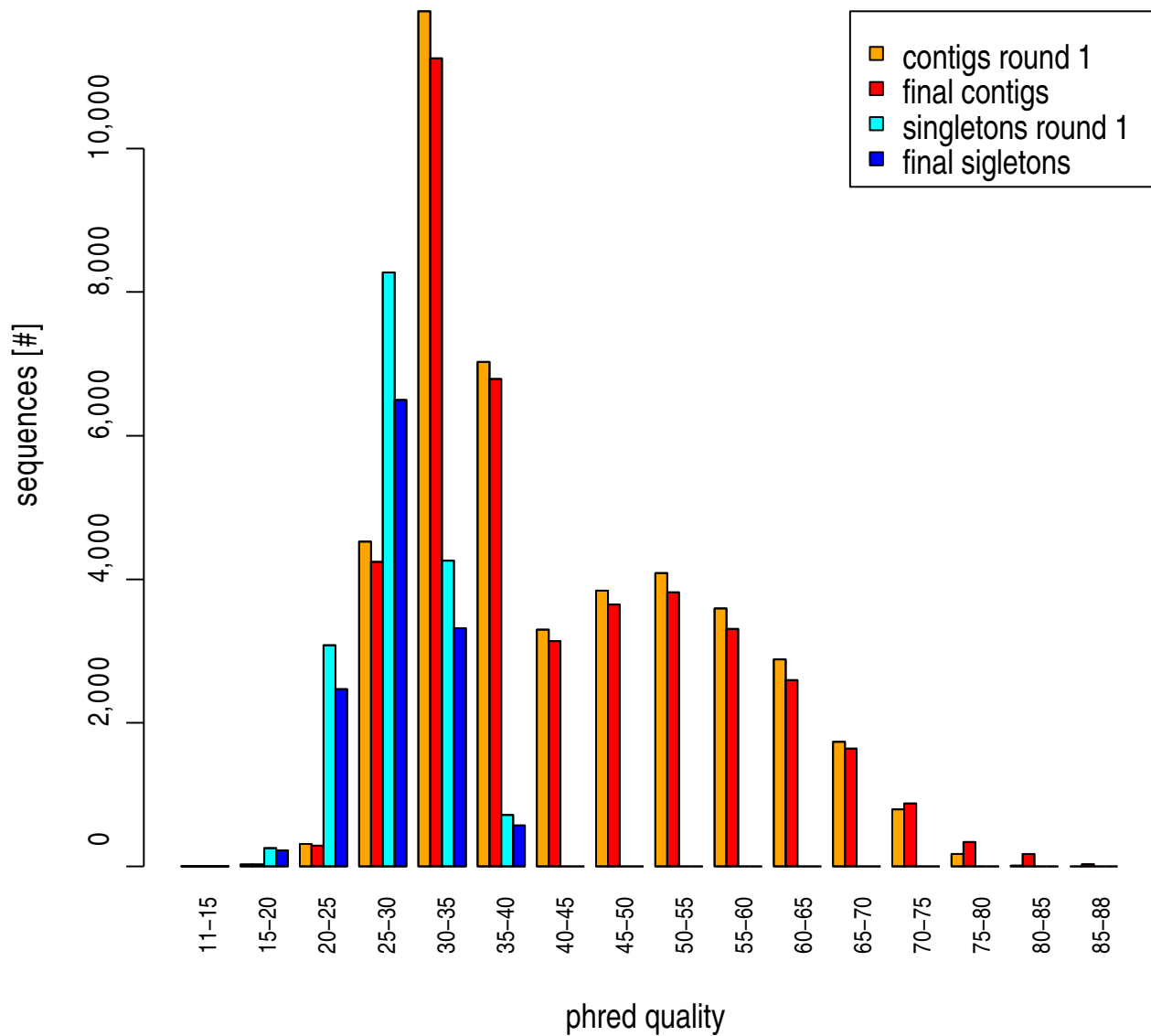

Supplement: Additional file 3 — Distribution of contigs' and singletons' average quality for first round and final assemblies. The figure shows how the number of singletons and contigs resulting from the first assembly (largest contig 2,732, N50 contig size 489, N90 contig size 324, N95 contig size 258), is reduced in the final set. [file 1471-2164-14-407-S3.pdf]

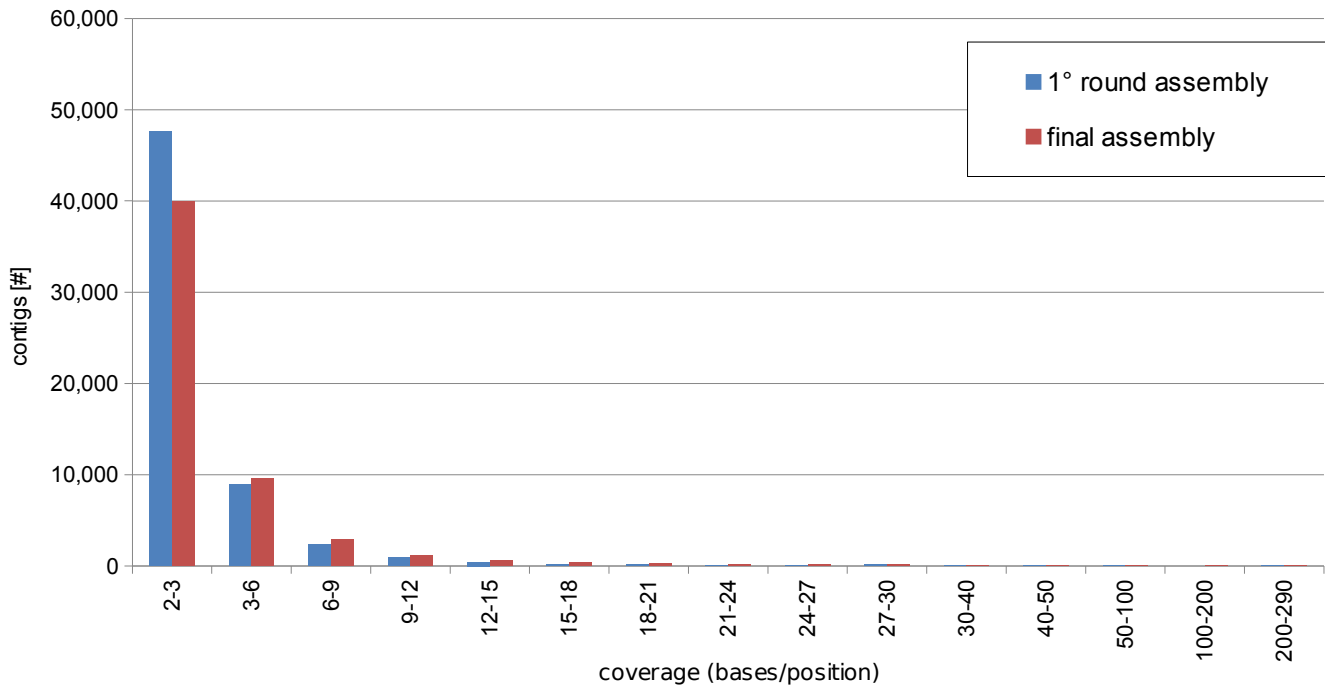

Supplement: Additional file 4 — Mean contigs coverage distribution for the first and final assemblies. Percentage of contigs falling in the different coverage intervals are referred to the final assembly. The average coverage of the contigs is quite low. As shown on the graph where about 61% of contigs have average coverage of only up to 3 per base. [file 1471-2164-14-407-S4.pdf]

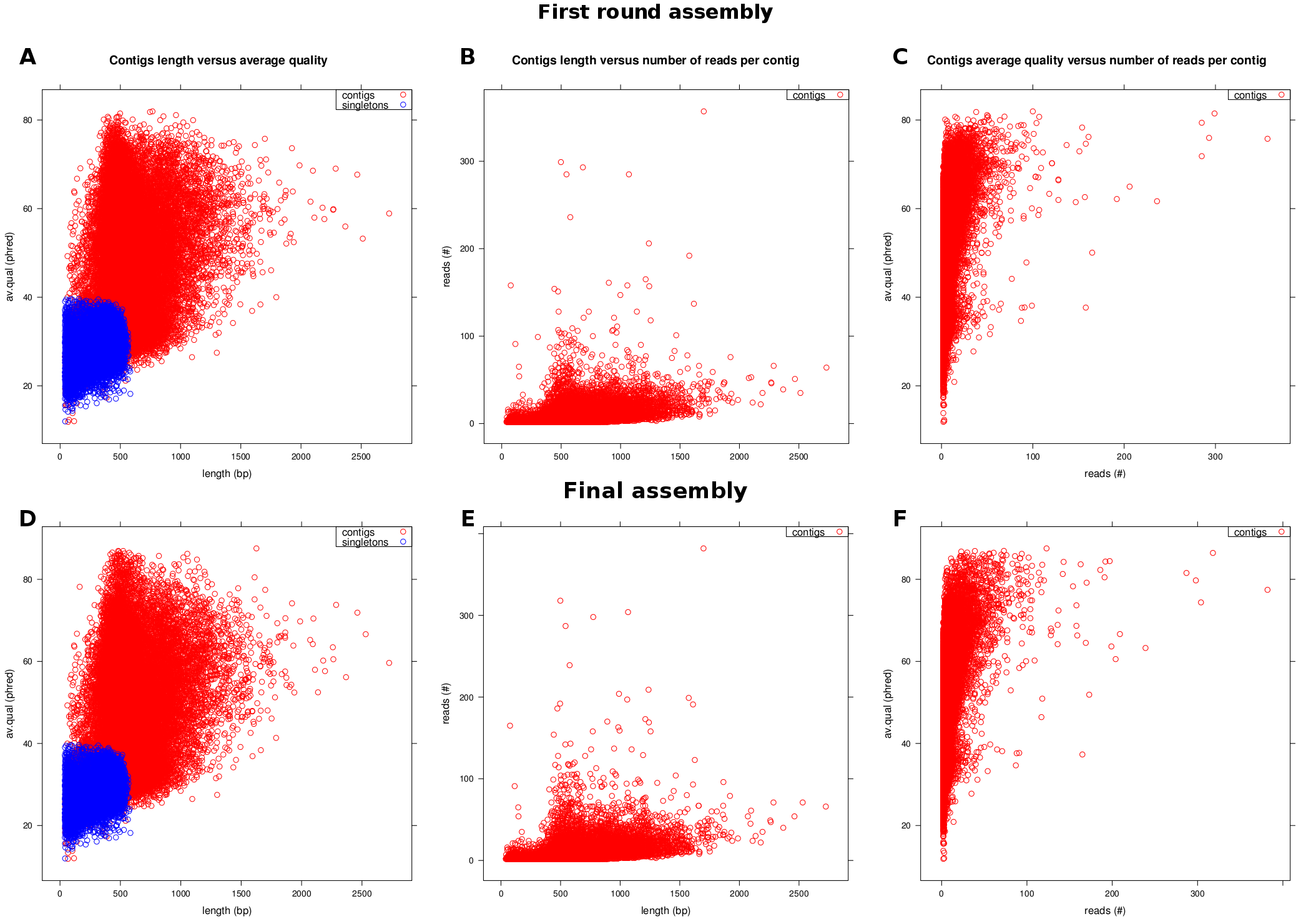

Supplement: Additional file 5 — Pair-wise relationships between main properties characterising total contigs obtained by the first and final assemblies. Pair-wise relationships between lengths and qualities (A, D), lengths and number of reads per contig (B, E), qualities and mean reads per contig (C, F), in the set of 60,825 contigs from the first assembly and 55,282 contigs from the (second) reassembly of the A. naccarii transcriptome. [file 1471-2164-14-407-S5.png]

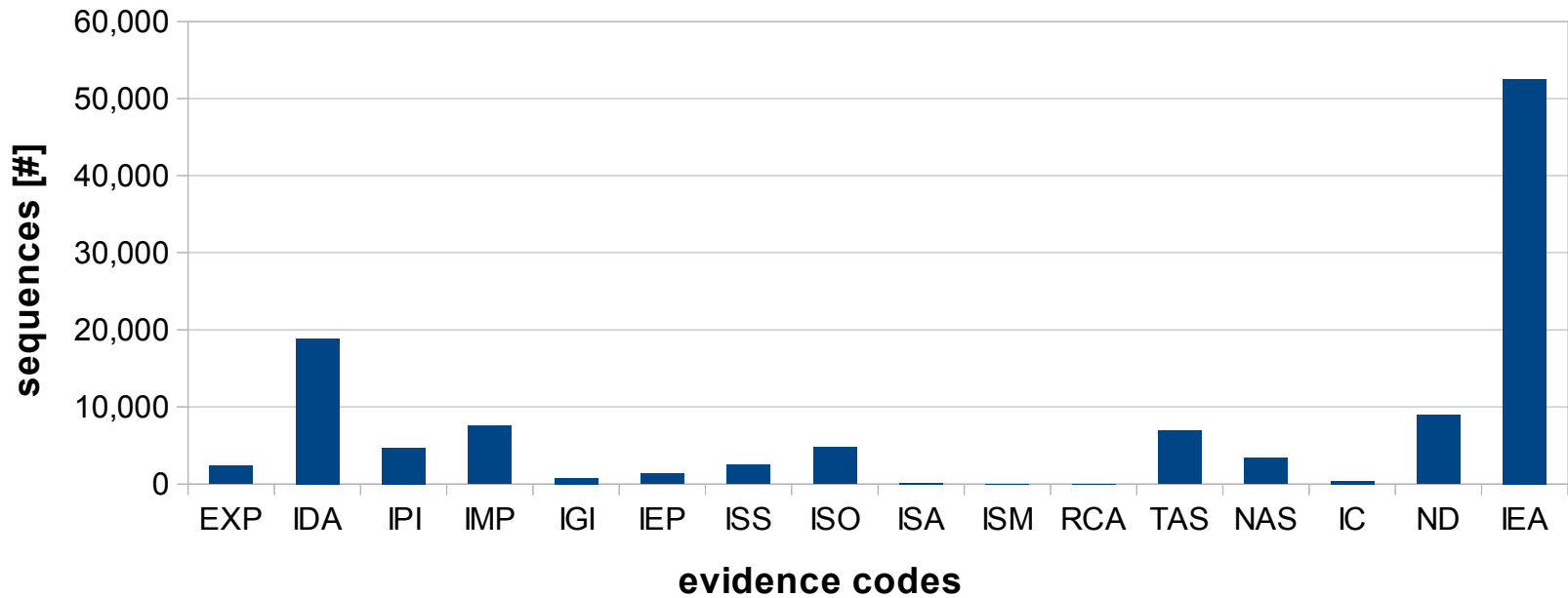

Supplement: Additional file 6 — Evidence code distribution of the annotation of A. naccarii transcriptome. Only the evidence codes assigned to at least one sequence are reported. EXP: Inferred from Experiment, IDA: Inferred from Direct Assay, IPI: Inferred from Physical Interaction, IMP: Inferred from Mutant Phenotype, IGI: Inferred from Genetic Interaction, IEP: Inferred from Expression, Pattern, ISS: Inferred from Sequence or Structural Similarity, ISO: Inferred from Sequence Orthology, ISA: Inferred from Sequence Alignment, ISM: Inferred from Sequence Model, RCA: inferred from Reviewed Computational Analysis, TAS: Traceable Author Statement, NAS: Non-traceable Author Statement, IC: Inferred by Curator, ND: No biological Data available. [file 1471-2164-14-407-S6.pdf]

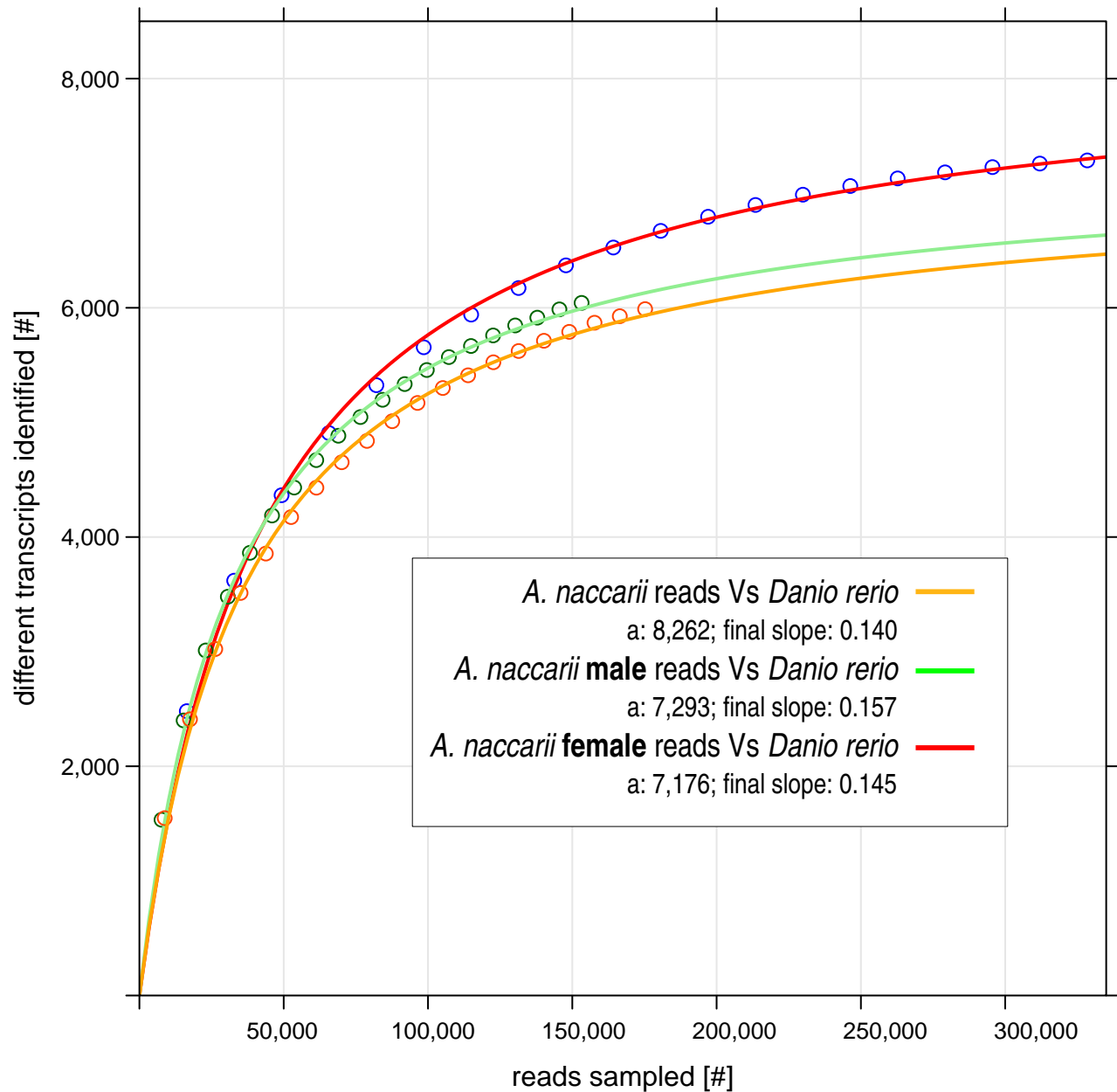

Supplement: Additional file 8 — Saturation curve for male, female and both A. naccarii cDNA libraries. Read subsets of increasing sample size were randomly extracted from total pool in the library. For each subset, contigs in which reads where assembled were identified. Each contigs pool was used to identify Danio rerio cDNAs (TBLASTX 2.2.25+ e-value 1e-03). Re-sampling and identification process was repeated 10 times for each sample size. A mean value and a confidence interval for the number of identified Danio cDNAs was calculated for each sample size. Hyperbolic model y = (ax)/(b + x) was fitted on points given by sample size versus average cDNAs hit so that model parameters “a” and “b” were estimated. The legend shows estimated parameter values obtained by fitting the hyperbolic model on the data. As can be seen, the curves from the single libraries retain the same trend and the difference is mostly due to the different number of reads in each library. [file 1471-2164-14-407-S8.pdf]

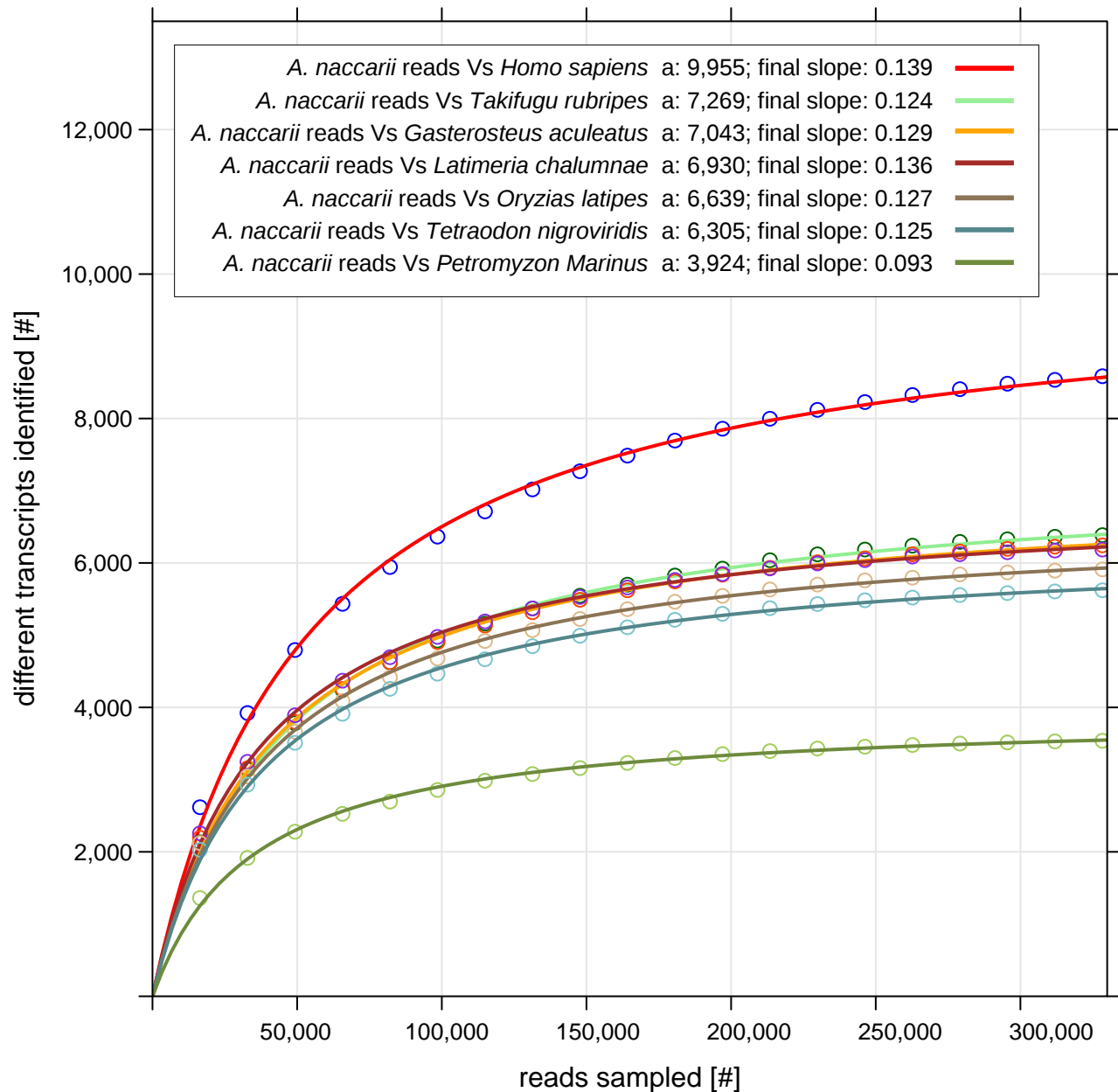

Supplement: Additional file 9 — Saturation curves plot for joint A. naccarii cDNA libraries against cDNA sets from other fishes. Were constructed several saturation curves, starting from total reads from the two libraries and using different sets of cDNA as a references. cDNA sets used are derived from all transcripts from Ensembl 66 for species in RS-list. The estimated parameters of the curves are reported in the legend. [file 1471-2164-14-407-S9.pdf]
